# Supplementary material for: The cardiopharyngeal mesoderm contributes to lymphatic vessel development in mouse
Source: eLife. 2022 Oct 5;11:e81515. doi: 10.7554/eLife.81515 (PMC9560160; doi:10.7554/eLife.81515)
Supplement: Supplementary file 1. [file elife-81515-supp1.docx]

**Supplementary File 1**

|  | Forward(5'-3') | Reverse(5'-3') |
| --- | --- | --- |
| *Rosa26^eYFP/+^* KI | AAAGTCGCTCTGAGTTGTTAT | AAGACCGCGAAGACTTTGTC |
| *Rosa26^eYFP/+^* WT | AAAGTCGCTCTGAGTTGTTAT | GGAGCGGGAGAAATGGATATG |
| *Rosa26^tdTomato^* KI | GGCATTAAAGCATATCC | CTGTTCCTGTACGGCATGG |
| *Rosa26^tdTomato^* WT | AAGGGAGCTGCAGTGGAGTA | CCGAAAATCTGTGGGAAGTC |
| Cre | ACATGTTCAGGGATCGCCAG | TAACCAGTGAAACAGCATTGC |
| *Prox1-flox* | CAGCCCTTTTGTTCTGTTGGCCAG | GCAGATGCTGTCCCTACCGTCC |
